# Books and Pamphlets Received

**Published:** 1886-08-20

**Authors:** 


### BOOKS AND PAMPHLETS RECEIVED.

*A Manual of Microscopical Technology* for Use in the Investigations of Medicine and Pathological Anatomy. By Dr. Carl Friedlaender, Lecturer on Pathological Anatomy in the University of Berlin. Translated by Stephen Yates Howell, M. A., M. D., of Buffalo, N. Y. New York and London: G. P. Putnam's Sons, Publishers.

This is not only an authority, but a most valuable guide, in Microscopical researches. No physican now-a-days can lay claim to the reputation of a diagnostician if he disregards the incursions, steady progress, and wonderful discoveries which microscopical technology has of late effected in the field of medical lore. Ignorance of the microscope, and of its potential revelations, is an *unpardonable sin* in the modern physician. We advise our readers to procure a copy of the above valuable work.

*A Manual of Diatetics.* By J. Milner Fothergill, M. D., Edinburgh, Physician to the City of London Hospital for Diseases of the Chest (Victoria Park); Honorary M. D. Rush Medical College, Chicago, Ill.; Foreign Associate Fellow of the College of Physicians, Philadelphia. 8 vo., extra muslin. 255 pages. Price, $2.50. New York: William Wood & Co.

This work treats, in an able and interesting manner, of the objects of Food; forms of food; methods of preparing food; beverages, condiments, stimulants; fluid food; artificial digestive agents; food in infancy; food in adult life; food in advanced life; food in acute diseases; food in convalescence; food in struma; food in anæmia; food in phthisis; food in heart and lung diseases; in albuminuria, in Bright's disease, in diabetes, in gout, in chronic invalids, in indigestion; food in biliousness; food in obesity, etc. The work would certainly be a very instructive and valuable addition to the library of the practitioner.

*A Compend of Pharmacy.* By F. E. Stewart, M. D., Ph. G., Quiz Master in Chemistry Pharmacy, etc., in the Philadelphia College of Pharmacy; Lecturer on and Demonstrator of Pharmacology in the Medico-Chirurgical College of Philadelphia; Demonstrator of Pharmacy in the Woman's Medical College of Pennsylvania; Member of the American Pharmaceutical Association. Based upon Prof. Joseph P. Remington's Text book of Pharmacy. Philadelphia: Blackiston, Son & Co. 1886.

The above is a very valuable compend, and presents the facts of Pharmacy in a form most easily comprehended and most readily remembered—useful especially to the student and to any one desiring to post up on pharmaceutical science.

*Hand-book of Practical Medicine.* By Dr. Herman Erchhorst, Professor of Special Pathology and Therapeutics, and Director of the University Medical Clinic in Zurich. Vol. I. Diseases of the Circulatory and Perspiratory Apparatus. One hundred illustrations. New York: William Wood & Co. 1886.

The work before us is the first volume. There is no introductory or preface, and it is not stated whether or not another volume will complete it; but we presume one more volume only will be issued. This volume contains over 400 large octavo pages, and a cursory examination suffices to show that it is a well written and practical work. The part relating to diseases of the lungs is especially instructive and interesting, and the illustrations are beautiful. We commend the work to the profession.

*The Therapeutics of the Respiratory Passage.* By Prosser James, M. D. Lecturer on Materia Medica and Therapeutics at the London Hospital Medical College; Physician to the Hospital for the Diseases of the Throat and Chest; late Physician to the North London Consumption Hospital; Consulting Physician to the Children's Home Infirmary, Victoria Park, etc. New York: William Wood & Co. 1884.

The above work contains over 300 octavo pages, and is well written and neatly published. Dr. James, the author, is evidently a practical man, and his work must prove very interesting and useful to any who will carefully peruse it. The subjects treated of are, principally, Nutrition in relation to Therapeutics; Respiration; Food and Diet; the Proximate Principles of Food; Food Stuffs; Variations in the Digestive Process; Aliments as Remedies; Iron, Phosphorous, etc.; Aids to Digestion, Transfusion, Water, Diluents, Beverages, Exercise, and Rest; Alcohol, Denutrients, Antipyretics, Neurotics, Pneumatics, etc.

*The Popular Sneaker;* comprising fresh selections in Poetry and Prose, humorous, pathetic and patriotic, for reading clubs, school declamation, home and public entertainments; containing the selections published in the Reading Club. By George M. Baker. Boston: Lee & Sheppard. 1885.

This is a neatly gotten up volume of 127 pages. It contains a great variety of selections, evidently chosen with great care and with judicious care, and must prove very acceptable to reading clubs, schools, etc. It is indeed an interesting and entertaining book to any one who is fond of choice reading.

*Venereal Memoranda;* a Manual for the Student and Practitioner. By P. A. Morrow, M. D. New York: William Wood & Co.

The author states in his preface that he has endeavored to "boil down," so to speak, the material embodied in more voluminous works on Venereal, and present the essential facts and principles in the most compact form possible consistent with clearness. The scope of the work is, therefore, more comprehensive than is usual in books of this kind. After a careful reading of the work we can say that the designs above expressed have been carried out more successfully than in any similar work which has fallen into our hands. Here, in aphoristic form, we get in a nutshell all the essential facts contained in the numerous ponderous volumes on venereal diseases, with a large number of practical recipes for treatment. 332 pages. Price $1, which could not be better appropriated by any physician than in purchasing this "Pocket Manual."

*Cutaneous Memoranda.* By Henry G. Piffard, A. M., M. D., Clinical Professor of Dermatology, University of the city of New York; Consulting Surgeon to the Charity Hospital, and the Bureau of Out-door Relief, Bellevue Hospital. Third edition.

This is one of Wood's Pocket Manuals, and is certainly a most convenient and useful little work. It contains a number of excellent illustrations of some of the most interesting phases of skin disease, and, indeed, presents a valuable condensed outline of skin affections, with etiology, symptoms, diagnosis, treatment, etc.

